# The Book World of Medicine and Science

**Published:** 1907-06-29

**Authors:** 


### The Book World of Medicine and Science.

### THE OXFORD MEDICAL PUBLICATIONS.

The first volumes of this series which we have received are certain of the Oxford Medical Manuals.* They mark a new departure which has a special interest for this journal, seeing that the editors, Dr. Keogh Murphy and Dr. Sutherland, appear to be working on pretty much the same lines as we have in mind in developing a modern medical journal. In form, typography, paper, and attractiveness the books leave little to desire.

There are too many "medical series" on the market which have not justified their existence. Criticism, to be valuable, must be honest, and we are glad to be able to say that, quite apart from the format of the Oxford Medical Publications, which is excellent, the whole series is practical in aim and generally efficient in result. But for the general practitioner it is the matter that counts. The aim of this series—"to give the busy practitioner in search of a handy, concise and inexpensive set of manuals just the practical assistance and information he requires"—is a high one. It remains to be seen how far each volume has realised this in practice.

In "Diseases of the Larynx," by Harold Barwell, M.B., F.R.C.S., Mr. Barwell is eminently practical. He has carried condensation to the pitch of a fine art. This is proved by the exceedingly able manner in which he has treated the conditions of leprosy and scleroma—conditions notoriously mishandled by most text-books where the throat is concerned. Sound, up to date, and lucidly written, this book has only once been diffuse, perhaps, in the chapter on laryngeal tuberculosis.

The next volume, "Treatment of Disease in Children," by G. A. Sutherland, M.D., F.R.C.P., again succeeds in giving the maximum of information in the minimum of space. Student and practitioner alike will find many valuable hints in it on diagnosis and treatment. It is a mistake, therefore, that the series as a whole—and this volume is an example of it—have not given more attention to the index.

Mr. Percy Sargent's volume, "Surgical Emergencies" (Percy Sargent, M.B., F.R.C.S.), is open to the criticism that it is written primarily for the beginner. Too many books, no doubt, ostensibly written for the expert, are valueless for elementary students and specialists alike; but we are sorry that Mr. Sargent had not a higher opinion of his public. Still, as far as he goes, common sense and practical knowledge have not deserted him.

Dr. Adamson contributes a little volume on "Skin Affections in Childhood" (H. G. Adamson, M.D., M.R.C.P.), as modest in tone and style as it is sound in information and practical in teaching. Here we have the series at its very best.

The fifth volume, on "Heart Disease," written by Dr. Poynton, we have not seen. The sixth is "Practical Anæsthetics," by H. Edmund G. Boyle, M.R.C.S., as unattractive in style as it is sound in information. Mr. Boyle has decided rather to adopt an eclectic method than to contribute an original study of his own. In his volume, therefore, the student may find facts brought together and arranged in such a manner as to be useful to the man who has hardly the time to consult the original and often lengthy authorities. The volumes to follow are "Diseases of the Male Generative Organs," "Diseases of the Ear," and "Diseases of the Nose and Throat."

By way of summary we may add that the series as a whole, excellent in form and cheap in price, is eminently suitable for the practitioner, admirable for the student who has yet to learn the greater part of his work, and in the case of the volumes by Dr. Murphy and Dr. Sutherland almost impeccable. The connection of Professor Osler with these publications is a guarantee of high purpose and honest endeavour, and if the later volumes, which we await with interest, reach the standard of Dr. Sutherland and Dr. Adamson, volumes unsurpassed as they are for smooth style and literary condensation, the series will undoubtedly prove one of the most valuable additions to the library of the medical practitioner.

* "The Oxford Medical Manuals," edited by J. Keogh Murphy, M.D., and G. Sutherland, M.D. Vols. I., II., III., IV., and VI. Price 5s. net each. Published by Henry Frowde, Oxford University Press, and Hodder & Stoughton, Warwick Square, London, E.C.

### APHORISMS OF A MASTER.*

Of the same size as the "Oxford Medical Manuals," published by the same firms, and very well printed, Dr. Gee's book is easily read and easily mastered. We have rarely read a professional work with so much pleasure. The lectures and aphorisms it contains are clear, terse, and delightful. English is, unfortunately, a language unfamiliar to most scientific writers, and when we say that Dr. Gee's style recalls the style of Morton we are paying the author a compliment not likely to be wasted on so careful and intelligent a student of medical history as himself. Cerebral hæmorrhage is a hackneyed subject, but in the hands of Dr. Gee it has become an enthusiasm. These interesting little discursions into the domain of medical etymology—for the chapters are mostly short—are charmingly used to point a meaning or illustrate a phrase; and one lays down the book with a feeling similar to that of the student who finished one of those interminable mediæval romances with the words, "Beautiful but all too brief." On no account should this book be borrowed; it must be bought and kept for the little masterpiece it is.

* "Medical Lectures and Clinical Aphorisms." By Samuel Gee, M.D., F.R.C.P. (Henry Frowde, Oxford University Press, and Hodder and Stoughton, Warwick Square, E.C. 5s. net.)

---

"Functional Nervous Disorders in Childhood." By Leonard Guthrie, M.D., F.R.C.P. (Henry Frowde, Oxford University Press, and Hodder and Stoughton, Warwick Square, E.C. 7s. 6d. net.)

Dr. Guthrie's book is interesting and pleasant reading, and can be heartily commended to the practitioner who wishes to obtain a concise, clear account of the neurotic child, with his many peculiarities, ailments, and affections. Many of these lectures have been delivered at the Post-Graduate College, and the book is therefore useful to practitioners, even to students, apart from the narrow standpoint of examination purposes. The chapters on "Fears of Neurotic Children," "Enuresis and Cyclical Albuminuria," "Moral Failings," and "Chorea" are specially interesting. In the last the pathology of chorea is summarised and a very full account of treatment is given. As a handbook of the nervous affections of children not due to distinctive organic lesions the book is valuable.

